# Cyclic GMP modulating drugs in cardiovascular diseases: mechanism-based network pharmacology

**DOI:** 10.1093/cvr/cvab240

**Published:** 2021-07-16

**Authors:** Alexandra Petraina, Cristian Nogales, Thomas Krahn, Hermann Mucke, Thomas F Lüscher, Rodolphe Fischmeister, David A Kass, John C Burnett, Adrian J Hobbs, Harald H H W Schmidt

**Affiliations:** Department of Pharmacology and Personalised Medicine, School for Mental Health and Neuroscience, Faculty of Health, Medicine and Life Sciences, Maastricht University, Maastricht University, Universiteitssingel 50, 6229 ER Maastricht, The Netherlands; Department of Pharmacology and Personalised Medicine, School for Mental Health and Neuroscience, Faculty of Health, Medicine and Life Sciences, Maastricht University, Maastricht University, Universiteitssingel 50, 6229 ER Maastricht, The Netherlands; Department of Pharmacology and Personalised Medicine, School for Mental Health and Neuroscience, Faculty of Health, Medicine and Life Sciences, Maastricht University, Maastricht University, Universiteitssingel 50, 6229 ER Maastricht, The Netherlands; H.M. Pharma Consultancy, Enenkelstrasse 28/32, A-1160, Vienna, Austria; Royal Brompton & Harefield Hospitals, Heart Division and National Heart and Lung Institute, Guy Scadding Building, Imperial College, Dovehouse Street London SW3 6LY, United Kingdom; Center for Molecular Cardiology, Schlieren Campus, University of Zurich, Wagistreet 12, CH-8952 Schlieren, Switzerland; INSERM UMR-S 1180, Faculty of Pharmacy, Université Paris-Saclay, F-92296 Châtenay-Malabry, France; Division of Cardiology, Department of Medicine, Ross Research Building, Rm 858, Johns Hopkins Medical Institutions, 720 Rutland Avenue, Baltimore, MD 21205, USA; Department of Cardiovascular Medicine, Mayo Clinic, 200 First St. SW, Rochester, MN 55905, USA; William Harvey Research Institute, Barts and The London School of Medicine and Dentistry, Queen Mary University of London, Charterhouse Square, EC1M 6BQ, London, UK; Department of Pharmacology and Personalised Medicine, School for Mental Health and Neuroscience, Faculty of Health, Medicine and Life Sciences, Maastricht University, Maastricht University, Universiteitssingel 50, 6229 ER Maastricht, The Netherlands

**Keywords:** Guanylate cyclase, Natriuretic peptides, Nitric oxide, Cyclic GMP, Biomarkers

## Abstract

Mechanism-based therapy centred on the molecular understanding of disease-causing pathways in a given patient is still the exception rather than the rule in medicine, even in cardiology. However, recent successful drug developments centred around the second messenger cyclic guanosine-3′-5′-monophosphate (cGMP), which is regulating a number of cardiovascular disease modulating pathways, are about to provide novel targets for such a personalized cardiovascular therapy. Whether cGMP breakdown is inhibited or cGMP synthesis is stimulated via guanylyl cyclases or their upstream regulators in different cardiovascular disease phenotypes, the outcomes seem to be so far uniformly protective. Thus, a network of cGMP-modulating drugs has evolved that act in a mechanism-based, possibly causal manner in a number of cardiac conditions. What remains a challenge is the detection of cGMPopathy endotypes amongst cardiovascular disease phenotypes. Here, we review the growing clinical relevance of cGMP and provide a glimpse into the future on how drugs interfering with this pathway may change how we treat and diagnose cardiovascular diseases altogether.

## 1. Background

For decades, the number of approved drugs has been in decline, indicating fundamental problems with respect to the productivity and innovation in basic, translational, and industrial research.^1^ Potential reasons for this include, amongst others, the underlying concept of disease, which is mainly based on symptoms, an organ and its phenotypic function rather than on molecular pathways. Indeed, causal, mechanistic understanding of disease is still the exception and currently relevant primarily for monogenic diseases.^2^ Common and complex diseases are primarily treated based on their symptoms, on risk factors or markers; clearly, a low-precision approach evidenced by the high numbers needed to treat and low efficacy of currently available drugs.^3–5^ Cardiology is no exception to this, and given its many unmet needs, this represents one of the most important knowledge gaps in medicine.^3^

Therapeutic agents that modulate the second messenger cyclic guanosine-3′ -5′ -monophosphate (cGMP) seem to be one exception to this conceptual roadblock and may lead the way towards a different, mechanism-based approach to a variety of diseases using also the powers of big data, networks and systems medicine.^6,7^ cGMP modulators have emerged as one of the most promising compounds in recent cardiovascular drug discovery.^7^ This may be because they do not act only symptomatically but, at least in a subset of suitable patients, target a disease mechanism rather than alleviating symptoms or modulating risk factors.^7,8^ In contrast, current cardiovascular treatments, such as renin–angiotensin–aldosterone system (RAAS) blockade do not follow a pathomechanistic approach. RAAS-blockers are not chosen as a therapy because in a patient up-regulation of RAAS has been measured, but solely symptomatically because RAAS blockade causes vasodilation. This is not a mechanism-based, causal therapy. The same holds true for other commonly used therapies, such as beta-blockers and calcium channel antagonists.

Cyclic GMP modulating drugs are used in a broad set of cardiovascular symptoms and conditions, such as angina, myocardial infarction, heart failure, pulmonary hypertension (PH), hypertensive crisis, and erectile dysfunction.^9–14^ Furthermore, preclinical evidence suggests benefit in ischaemic stroke.^8^ In addition, cGMP-related biomarkers, natriuretic peptides (NPs), are used to monitor heart failure patients.^10^

**Figure 1:**
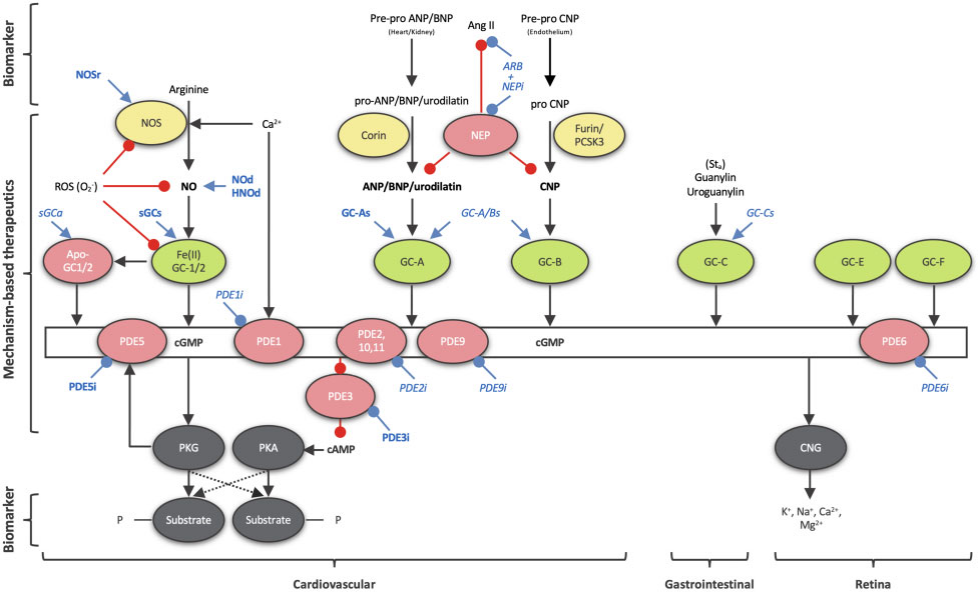
Classical, curated representation of cGMP signalling. Shown in green are the GCs and in yellow their positive regulators; NO produced by NOS for soluble GC and NPs (ANP, BNP, and CNP) for particulate GC. Negative regulators (cGMP metabolizing PDEs 1, 2, 5, 6, 9, 10, 11, and NPs degrading NEP) and pathophysiological conditions (oxidized/heme-free apo-sGC) are shown in pink. All clinically relevant cGMP-modulating drugs are shown in blue (bold for approved drugs and in italic for drugs under investigation): ARB, Angiotensin II receptor blockers; GC-As, GC-A stimulators; GC-A/Bs, GC-A/B stimulators; NEPi, neprilysin inhibitors; NOd, NO donors; NOSr, NOS recoupling nutraceuticals; sGCa, sGC activators; sGCs, sGC stimulators; PDEi, PDE inhibitors. cGMP effector proteins (PKG and cyclic nucleotide-gated ion channels, CNG) and their substrates are shown in grey. cGMP can also inhibit some isoforms of the PDE enzyme family. In turn, this leads to an altered phosphoprotein profile, a decrease in intracellular calcium levels and sensitivity, and altered cGMP and cAMP levels (proffering crosstalk between cGMP and cAMP networks). GC-C is localized in intestines and GC-E and -F in the retina, thus not relevant to the context of this review. Ang II, angiotensin II; Sta, heat-stable enterotoxin I STa.

In the cardiovascular system, the effects of cGMP are predominantly mediated by cGMP-dependent protein kinases and cGMP-regulated phosphodiesterases (PDEs)^15^ (*Figure 1*). Cyclic GMP appears to exert almost exclusively beneficial effects with a single overt limitation, vasodilation which in some patients may lead to hypotension and syncope, in particular in combination therapy.^16^ Therefore, cGMP increase leverages apparently only additional therapeutic gain, particularly in those cardiovascular conditions associated with a proven, i.e. mechanism-based, deficit in cGMP signalling. Clinically, this is achieved mainly by two approaches, either by (i) activating guanylyl cyclases to increase cGMP synthesis or by (ii) inhibiting relevant PDEs to inhibit cGMP breakdown. Future cGMP-centric strategies will most likely include combinations of different types of cGMP-modulating drugs and be increasingly guided by additional innovative plasma- or cell-based biomarker panels yielding powerful therapeutic and diagnostic (‘theranostic’) couples with cGMP-modulating drugs for cardiovascular precision medicine.

## 2. Cyclic GMP, a mechanism-based approach for cardiology

cGMP-modulating drugs are very promising in cardiovascular medicine and provide a broad clinical applicability. Indeed, cGMP-modulating drugs provide protective effects within the heart and vasculature by inhibiting vascular smooth muscle contraction and proliferation, suppressing platelet and leukocyte reactivity, and both anti-fibrotic and anti-hypertrophic actions.^15^ A second, probably even more promising aspect is the fact that dysfunctional cGMP formation and signalling appear to play direct pathomechanistic roles in cardiovascular disease. Genome-wide association studies have identified single nucleotide polymorphisms in genes encoding several components of this pathway to be correlated with cardiovascular diseases (*Table 1*).^17,18^ Thus, any up-regulation of cGMP has the potential to act and cure in a unique mechanism-based manner.^8,15^*Table 2* demonstrates cGMP-related drugs for therapeutic cardiovascular applications, either approved or under clinical investigation.

**Table 1:** cGMP-related loci. Identified to be associated with cardiovascular diseases by genome-or exome-wide association studies. NPPA genetic variant rs5068 and GUCY1A3 variant α1-A680T may protect against metabolic syndrome and PH, respectively

Gene	Chromosome	Association with
ANP [*NPPA*]	1	AF^23^, BP^17^, MetS^24^, VR^178^
BNP [*NPPB*]	1	BP^17^
Furin [*PCSK3*]	15	BP^17^, MI/CAD^179^
eNOS [*NOS3*]	7	BP^180^, CAD^181^, MetS^19^
NPR1 *[NPR1]*	1	BP^22^, VR^178^
PDE5A *[PDE5A]*	4	CAD^18^
sGCα_1_ [GUCY1A3]	4	BP^17,182^, PH^183^
sGCα_1_ [*GUCY1A3*] + CCTη [*CCT7*]	4+2	MI/CAD^21^
sGCβ_1_ [*GUCY1B3*]	4	BP^17,182^

AF, atrial fibrillation; BP, blood pressure; CAD, coronary artery disease; CCT7, chaperonin containing TCP1 subunit 7; MetS, metabolic syndrome; MI, myocardial infarction; PH, pulmonary hypertension; VR, ventricular remodelling.

**Table 2:** cGMP-modulating drugs. NO donors, NOS targeting compounds, soluble GC (GC-1/2) stimulators and activators, GC-A/B stimulators, NEP inhibitors, and PDE inhibitors, either approved or under clinical investigation for therapeutic cardiovascular applications

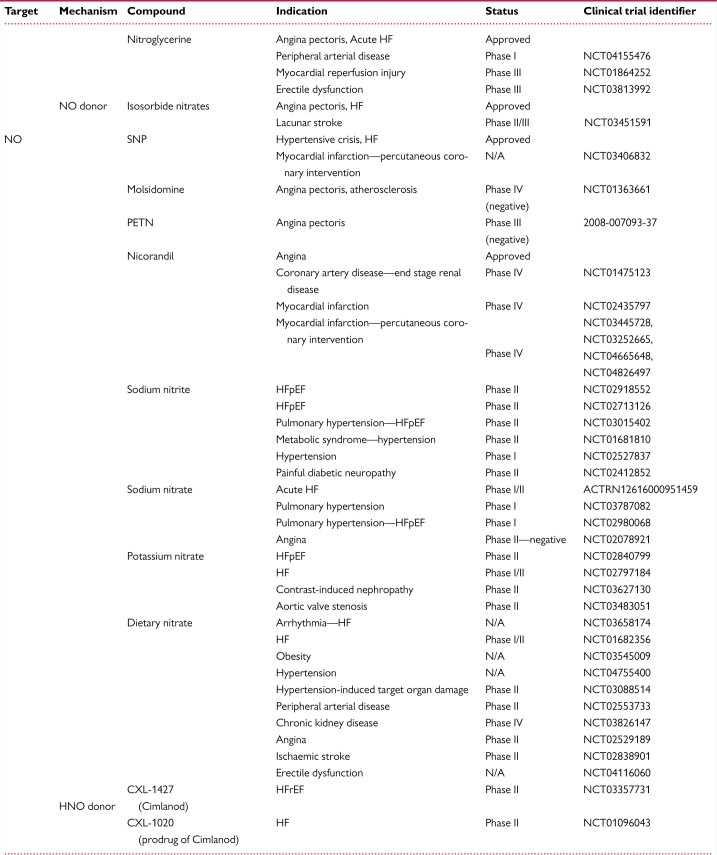
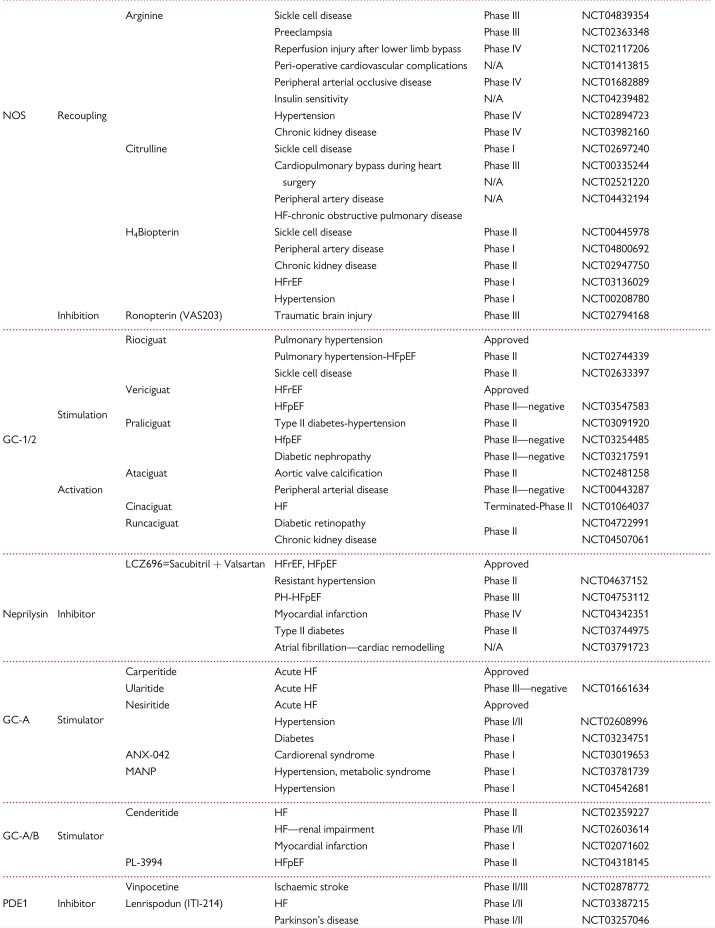
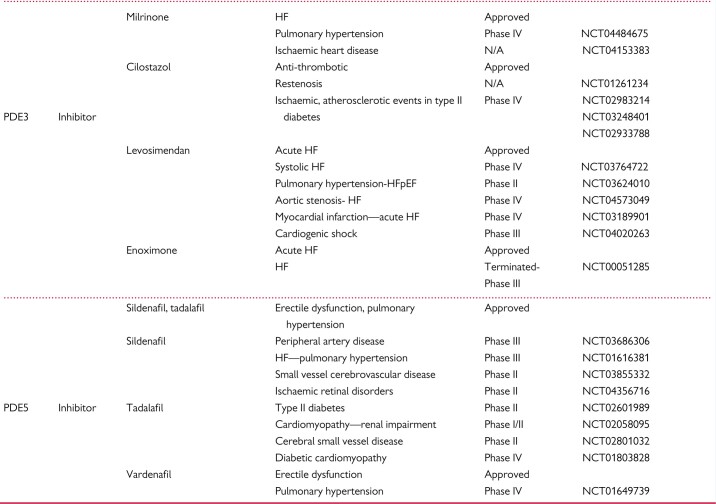

### 2.1 cGMPopathies

cGMPopathies describe a dysregulation of cGMP signalling by reduced cGMP synthesis, increased cGMP breakdown, or defective cGMP downstream signalling.^7^ This term is a part of an overall new approach to disease namely, not to define diseases based on a symptom in an organ but by a causal mechanism. This mechanism may lead to symptoms in different organs. Coronary artery disease (CAD), myocardial ischaemia (MI), heart failure (HF), hypertension, diabetic nephropathy, and metabolic syndrome belong to cGMPopathies.^15,18,19^*NOS3* polymorphisms have been associated with CAD/MI, hypertension, metabolic syndrome, diabetes mellitus, and diabetic nephropathy;^18,19^ a shorter half-life variant of NOS3 with reduced event-free survival in HF with systolic dysfunction;^20^ a *PDE5A* variant with CAD;^18^ genetic variants of *GUCY1A3* with blood pressure, CAD and MI.^18^ Mutations in the α1-soluble guanylate cyclase (sGC) subunit and CCT7η encoding genes, leading to decreased sGC activity, have been associated with myocardial infarction risk.^21^ A deletion mutation in the type A human NP receptor gene was associated with essential hypertension and ventricular hypertrophy.^22^ In families with atrial fibrillation, a frameshift mutation in the atrial natriuretic peptide (ANP) encoding gene has been identified and possibly involved in the development of the disease.^23^ Moreover, the ANP (*NPPA*) genetic variant rs5068 characterized by increased production of ANP in humans has demonstrated a phenotype of lower blood pressure, reduced risk of hypertension, and decreased prevalence of metabolic syndrome.^24^ The genetic involvement of cGMP signalling in these diseases is further confirmed by pathophysiological data.

Endothelial dysfunction characterized by NO dysregulation and inflammation coincides with reactive oxygen species (ROS) formation leading to cardiovascular disease states.^25^ Notably in heart failure, neurohumoral activation, secretion of inflammatory messengers, and altered shear stress lead to ROS generation that interferes with NO.^26^ The resulting endothelial dysfunction causes a further imbalance of NO and unphysiological ROS formation that worsens HF.^26^ Reduced PKG activity and cGMP concentrations, probably resulting from low NO bioavailability, are related to cardiomyocyte stiffness in the HF with preserved ejection fraction (HFpEF) myocardium.^27^ Higher levels of uncoupled eNOS and PDE9A were also shown in HFpEF myocardium.^28,29^ Elevated ANP levels have been associated with HF, but blunted responses to ANP infusion in HF patients indicate the possibility of down-regulation of ANP receptors^30^ or up-regulation of the NP-metabolizing receptor.^31^ In human failing hearts, guanylate cyclase (GC)-A in cardiomyocytes does not respond to ANP stimulation,^32^ and PDE1C and PDE5 levels are up-regulated.^33,34^

Endothelial dysfunction, as a result of dysregulated ROS formation and inflammation, correlates with atherosclerosis.^35^ Increased activity of NADPH oxidase (as a source of superoxide) was associated with decreased endothelial vasorelaxations and increased atherosclerotic risk factors.^36^ In CAD patients, the oxidized form of sGC was increased^37^ and asymmetric-dimethyl-L-arginine (ADMA) levels associated with eNOS uncoupling.^38^ In hyperlipidaemia, cGMP modulators are unable to induce cardioprotective effects, suggesting a dysfunction downstream of cGMP formation.^39^ In the same study, PKG activity is down-regulated in hyperlipidaemic rats as assessed by troponin I phosphorylation.^39^

Nitric oxide is implicated in impaired vasodilation in hypertensive patients.^40^ Endothelial NO production by eNOS is decreased and systemic NO production by iNOS increased (resulting in hyperproduction of toxic NO levels) in patients with coronary heart disease (CHD) associated with hypertension; these effects are more expressed in CHD with hypertension compared to isolated CHD patients.^41^ In addition, diminished L-arginine transport has been proposed as a link from dysfunctional NO signalling to essential hypertension.^42^ In pulmonary arterial hypertension (PAH) patients, arginine levels in airway epithelial cells are inversely associated with pulmonary arterial pressures, while in pulmonary artery endothelial cells NO production is reduced and arginase activity higher.^43^

Importantly, when moving from organ- and symptom-based to mechanistic disease definitions not all patients with a given clinical disease phenotype is expected to suffer from the same cause. cGMPopathy rather represents one endotype, and there will be others that could lead to a similar phenotype. NOX5-induced uncoupling of eNOS as a causal mechanism of age-related hypertension is a good example of this as it affects approximately only one in four or five patients with hypertension.^44^ Different endotypes of one phenotype may have different symptoms or comorbidities, which in multiscale modelling is used to identify the mechanism of unclear endotypes.

## 3. Drugs increasing cGMP generation

### 3.1 Nitric oxide and its receptors, GC-1 & GC-2

The traditionally defined ‘soluble guanylate cyclase (sGC)’, more recently termed guanylyl cyclases GC-1 and GC-2 (to differentiate them from the membrane-spanning, guanylyl cyclases, GC-A, GC-B, activated by NPs), is a heterodimeric haemoprotein comprised of one of two alpha subunits (α_1_ or α_2_) and a beta subunit (β_1_). An N-terminal pocket binds Fe(II)haem via a proximal histidine and thereby confers sensitivity to NO.^45,46^ Binding of NO cleaves the proximal histidine-Fe(II) haem bond and induces a structural shift that activates the catalytic site converting GTP into cGMP.^45,46^ Inappropriate formation of ROS, in particular superoxide, can interfere with NO-cGMP signalling in at least three ways: (i) by chemically scavenging NO; (ii) by uncoupling NO synthase (NOS); or (iii) by oxidizing the haem group within GC-1/2 from Fe(II) to Fe(III) eventually resulting in heme-deficient apo-GC. The latter is not only insensitive to NO but also prone to rapid degradation.^47^

Therapeutically, three avenues are clinically promising for reinstating or augmenting NO-GC-1/2 signalling: (i) repairing or replacing NO synthesis; (ii) sensitizing GC-1/2 to lower levels of NO by allosteric modulator compounds, so-called sGC stimulators;^48^ or (iii) re-activating NO-insensitive, haem-free apo-GC by haem-mimetics, so-called sGC activators, which also prevent enzyme degradation.^48,49^

#### 3.1.1 Repairing or replacing NO synthesis

Recoupling NOS, by dietary supplementation of its redox-sensitive cofactor tetrahydrobiopterin or its substrate L-arginine, is pre-clinically effective. So far, there are, however, no clinical trials with positive outcomes to demonstrate the efficacy of such a nutraceutical approach.^50^ Therapeutically, NO substitution with so-called NO-donor or nitrovasodilator compounds has the longest history (e.g. in angina, heart failure), but also limitations, such as pharmacokinetic^51^ and pharmacodynamic^52^ tolerance, which requires therapy-free intervals to regain nitrate sensitivity. According to ESC/AHA guidelines, the use of sodium nitroprusside, chemically an NO^+^ donor, is limited to i.v. application in hypertensive emergencies e.g. as first-line treatment in acute cardiogenic pulmonary oedemas, and in acute HF as second-line therapy.^10,14,53,54^ Short-acting nitrates, such as nitroglycerine and isosorbide dinitrate (ISDN), can be used as first-line therapy for pain relief of an angina attack, whereas long-acting nitrate formations of nitroglycerine, ISDN, or isosorbide mononitrate (ISMN) are used as second-line treatments for angina prophylaxis.^9,12,55,56^ Nitroglycerine and ISDN are also considered second-option vasodilators in acute HF.^10,54^ A combination of ISDN and hydralazine can be used as second-line therapy in HF with reduced ejection fraction (HFrEF).^10,54^ Additionally, molsidomine, an NO-donor upon metabolism, is an antianginal drug; but not yet recommended in routine use.^57^ Three NO donors, pentaerythritol tetranitrate, nicorandil and nitroxyl (HNO or NO^−^), seem to be devoid of tolerance, which awaits to be exploited therapeutically.^58,59^ However, PETN is not recommended for stable angina yet due to not sufficient efficacy evidence.^58^ Nitroxyl donors, such as BMS-986231 (previously CXL-1427) differ from pure NO donors and showed a favourable safety and haemodynamic profile in acute decompensated HF.^60^ Nicorandil, a nicotinamide- nitrate ester and K+ channel opener, is suggested as a second-line antianginal drug for patients with chronic coronary syndromes in Europe but not approved in USA.^9,10,12,58^ In addition to nitrate tolerance, a general concern is that under conditions of elevated ROS levels, NO donors may lead to unwanted reactive nitrogen species and endothelial dysfunction.^51,61^

Two more targeted and mechanism-based strategies circumvent these shortcomings and risks i.e. sGC stimulators and sGC activators. Despite their very similar sounding names, they have distinct targets, i.e. Fe(II)haem-containing GC-1/2 and apo-GC-1/2, respectively. Importantly, both enhance cGMP synthesis independently of modulating NO levels and are thus devoid of tolerance.^48^

#### 3.1.2 sGC stimulators

These compounds interact with an allosteric site to sensitize (FeII)haem containing GC-1/2 for NO.^46^ If tissue levels of NO are low, this will result in a mechanism-based ‘recovery’ of a physiological cGMP response. However, if levels of NO are high, these compounds have limited or no additional effect on cGMP.

Riociguat (BAY 63–2521) was the first registered sGC stimulator approved for use in PH, i.e. chronic thromboembolic pulmonary hypertension and PAH.^62^ No evidence-based first-line therapy is suggested for PH, but riociguat is one of the initial monotherapies that can be chosen according to ESC/CHEST guidelines.^11,63^ However, following the early termination of the phase II RISE-IIP trial because of serious adverse events, riociguat is not suggested to patients with PH associated with idiopathic interstitial pneumonia.^64^ Riociguat was also evaluated in PH associated with left systolic heart failure, and, despite not meeting the primary endpoint of change in mean pulmonary artery pressure (mPAP), it had favourable effects on secondary outcomes.^65^ The DILATE-1 trial tested riociguat in patients with HFpEF and PH; stroke volume and cardiac index were increased, systolic blood pressure and right ventricular end-diastolic area decreased, but there was no significant change on peak decrease in mPAP.^66^ At the moment, riociguat is under investigation for its long-term treatment in PH associated with HFpEF (NCT02744339).^67^

Vericiguat (BAY-1021189) reached the primary outcome in reducing cardiovascular mortality or hospitalization for HF in a Phase 3 clinical trial for HFrEF (VICTORIA) and recently received approval in USA.^68,69^ It was also further evaluated in a phase IIb HFpEF trial (VITALITY-HFpEF) where it failed to improve the quality of life (physical limitation score of the KCCQ), which was the previously suggested beneficial outcome in phase IIb SOCRATES-PRESERVED.^70,71^

Another sGC stimulator with promising effects in an animal model of cardiorenal failure, praliciguat (IW-1973), showed favourable trends in metabolic and hemodynamic variables in patients with type 2 diabetes (T2D) and hypertension.^72,73^ However, it failed to reach the primary endpoints of improved peak rate of oxygen consumption and reduction in albuminuria in Phase 2 trials for HFpEF and diabetic nephropathy, respectively.^74,75^

A shortcoming of all these trials still is that they stratified patients purely on clinical grounds and did not use biomarkers to identify HFpEF and HFrEF patients with a mechanistic endotype indicating cGMP dysregulation. By failing to do so, potential benefits in some patients may have been diluted through non-responders with different underlying pathomechanisms. Of note, the terms HFpEF and HFrEF are purely descriptive overarching terms, recently complemented by Heart Failure with mid-range or intermediate ejection fraction (HFmrEF or HFiEF).

#### 3.1.3 sGC activators

These molecules specifically bind to the NO-insensitive, haem-free or -oxidized apo-GC-1/2.^48^ Large molecules, such as cinaciguat (BAY58–2667), but not the smaller ataciguat (HMR1766) binding the oxidized form,^76^ occupy the empty haem site and prevent its ubiquitination and proteasomal degradation,^47^ thereby both an activating and stabilizing apo-sGC. However, clinical phase II trials (COMPOSE programme) with cinaciguat in patients with acute heart failure had to be stopped prematurely due to severe hypotension.^77^ Moreover, the safety of ataciguat (HMR1766) has been evaluated in patients with moderate aortic valve stenosis (NCT02049203) and efficacy in patients with aortic valve calcification (NCT02481258) and peripheral arterial disease (NCT00443287); however, ataciguat’s development was discontinued.^78^ A novel compound with improved physicochemical and pharmacokinetic characteristics, runcaciguat, is now investigated in chronic kidney disease and diabetic retinopathy.^78^

### 3.2 NPs and their GC-coupled receptors

**Figure 2:**
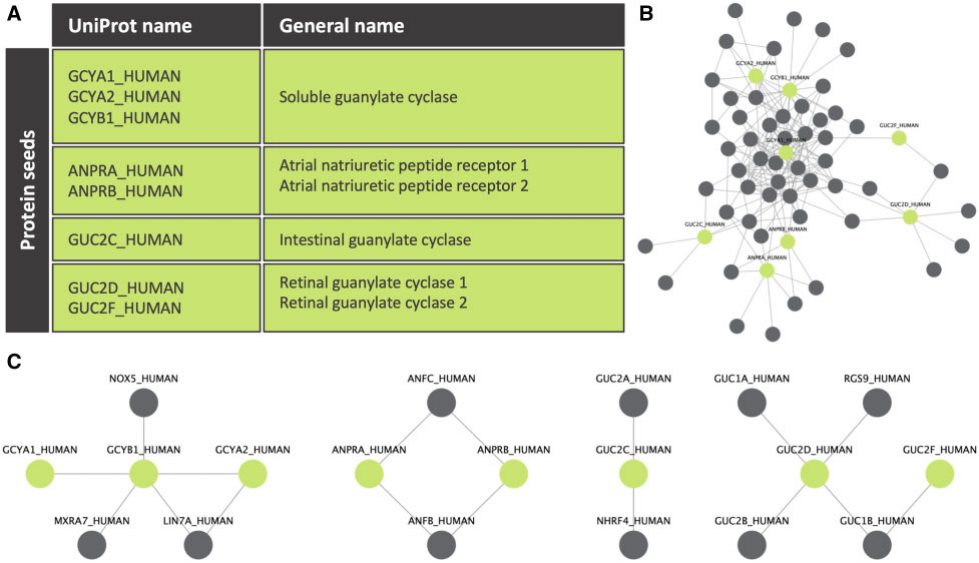
Unbiased, PPI-based GCs signalling modules. (*A*) Table of clinically relevant GCs. These were used as seed proteins to start building their first neighbour PPI network. (*B*) First neighbour PPI network of GCs from IID. Seed proteins are shown in green and their first neighbour PPI interactions in grey. A line was drawn between two proteins when they interact with each other according to IID. Only experimentally validated data were used. (*C*) Four different modules are extracted after curating the network with a 0.1 subnetwork participation degree (SPD) cut-off. The SPD cut-off removes highly connected and non-relevant proteins, thus, confirming *in silico* the literature function- and organ-based distinction of different GCs.

The second cGMP forming family is plasma membrane-spanning GCs, often referred to as particulate GCs due to their subcellular localization in the particulate fraction. They comprise seven members (GC-A to GC-G), of which two, GC-D and GC-G, are pseudogenes and three, GC-C, GC-E, and GC-F, are—as far as we know—not relevant for the cardiovascular system.^79,80^ This organ- and function-based GCs compartmentalization is further confirmed *in silico* (*Figure 2*). Here, we make use of experimentally validated protein–protein interaction (PPI) data from the Integrative Interactive Database (IID).^81^ Starting from GC-coupled receptors, we look at their direct protein interactions in IID to build the first neighbour PPI network. After pruning the network from highly connected but non-relevant protein interactions, four different subnetworks or signalling modules are extracted: (i) sGC module, (ii) ANP receptors module, (iii) intestinal GC module, and (iv) retinal GCs module. GC-A and GC-B are homodimers containing an N-terminal extracellular ligand-binding domain for NPs.^79^ They are therefore also termed NP receptors NPR-A and NPR-B, respectively.

Humans express four types of NPs, atrial (ANP), brain (BNP), and C-type natriuretic peptide (CNP) and urodilatin.^82^ ANP, BNP, and urodilatin each activate GC-A; CNP is the sole endogenous GC-B agonist. Via GC-A/B, NPs have a wide range of cardio- and vaso-protective effects, i.e. natriuresis, diuresis, inhibition of vasoconstriction, as well as anti-hypertrophic, anti-fibrotic and anti-proliferative effects and possibly also metabolic actions, such as lipolysis and browning of adipocytes.^82^ NPs bind to another NP receptor, natriuretic peptide clearance receptor (NPR-C), which has no GC activity and is responsible for NPs clearance from the circulation. However, CNP activation of NPR-C plays a crucial role in cardiac function and vascular homeostasis.^83^

Elevated NP levels are also disease biomarkers, in particular in heart failure. Paradoxically though, increased expression and release of NPs does not necessarily translate into enhanced activation of the particulate GC-cGMP pathways. Instead, there appears to be a disconnect. In heart failure, proBNP, the precursor of mature BNP, is the predominant circulating form and lacks significant GC-A activating properties compared to BNP.^84^ More recently, studies have revealed the presence of glycosylation of ANP, resulting in a molecular form with reduced GC-A activation.^85^ The presence of altered molecular forms of ANP and BNP with reduced cGMP production supports the use of native and designer synthetic NPs to rescue these NP structural abnormalities. Such a hormone replacement strategy is also underscored by the presence of an ANP deficiency in human heart failure due to either reduced production and/or increased peptide degradation.^86^ Therefore, even when plasma levels of NPs are elevated in heart failure and other conditions, pharmacological GC-A/B stimulation may still be beneficial. Three therapeutic approaches to enhance NP signalling have entered the clinic; natural peptides, such as neseritide or carperitide, designer peptides, such as uralitide, and molecules that inhibit peptide breakdown via neutral endopeptidase (NEP), such as sacubitril.

#### 3.2.1 Recombinant and designer NPs

The clinical utility of GC-A/B-cGMP stimulation was first examined with recombinant ANP, carperitide, in acute heart failure,^87,88^ but its impact on in-hospital mortality and length of hospitalization was inferior to nitrates.^89^ LASCAR-AHF now tests the long-term effects of carperitide in acute HF.^89^ Despite the lack of sufficient evidence, carperitide is used in Japanese practice as second-line treatment in acute HF.^90^ In the J-WIND trial, recombinant ANP decreased infarct size and improved ejection fraction in patients with myocardial infarction undergoing percutaneous coronary intervention^91^ but had no effect on in-hospital mortality.^92^

Another NP, a synthetic form of urodilatin, ularitide, neither affected a clinical composite endpoint nor cardiovascular mortality in patients with acute HF.^93^ Similarly, the recombinant BNP, nesiritide, despite a small change in dyspnoea, neither improved all-cause death nor re-hospitalization for HF in patients with acute, decompensated HF.^94^ Even worse, a meta-analysis associated the use of nesiritide to an increase in the short-term risk of death in such patients.^95^ This perhaps provides a warning that excessive GC-A activation may be detrimental due to significant hypotension that may compromise renal function and lead to sympathetic activation, both unfavourable events in patients with heart failure. Currently, it is considered a second-line intravenous vasodilator for acute HF in Europe and USA.^10,54^

An alternative strategy has been the development of ‘designer’ NPs, which aim to combine beneficial effects of different endogenous peptides. CD-NP, i.e. cenderitide (CD-NP), is a modified CNP with 15 additional amino acids at the C-terminal tail of DNP (a related peptide identified in the venom of the green mamba, *Dendroaspis angusticeps*).^96^ The rationale behind this combination is to promote the vasodilator and anti-fibrotic properties of C-type natriuretic peptide (at least in part via GC-B stimulation), with the natriuretic properties of DNP (which stimulates GC-A but is thought to avoid a dose-limiting hypotension). Both these pharmacodynamics as well as safety, i.e. absence of hypotension, were established in stable HF patients.^97^ ANX-042, a peptide designed based on an alternative spliced variant of BNP, is currently under investigation as a non-hypotensive drug in cardiorenal syndrome (NCT03019653).^98^ Beyond heart failure, the designer ANP-analogue (MANP) was engineered as a novel ANP mimetic whose biological properties of natriuresis, blood pressure-lowering, and aldosterone suppression are greater than ANP.^99^ This analogue retains the 28 amino acids of ANP but possesses a novel 12 amino acid extension to the carboxyl terminus resulting in greater resistance to enzymatic degradation by neprilysin and reduced clearance by the NPR-C.^99^ MANP has been recently investigated in hypertension and metabolic syndrome, where it showed a safe profile, a borderline significant blood pressure decline and a significant increase of cGMP and non-esterified fatty acids levels.^100^

#### 3.2.2 NEP inhibitors

NEP (also known as neprilysin) is a membrane-bound metalloproteinase responsible for the breakdown of many vasoactive mediators, including NPs, but also glucagon, bradykinin, oxytocin, substance P, angiotensin II, endothelin, and beta-amyloid.^101^ Clinically, however, NEP inhibitors (NEPi) have little or no effect on blood pressure despite significantly elevated plasma NP concentrations.^102^ This paradox was attributed to the fact that NEP metabolizes both vasodilating (e.g. NPs, bradykinin) and vasoconstricting (e.g. angiotensin II and endothelin) peptides, thereby possibly outweighing any hemodynamic benefit.

As a result, drug development in this area focused on a combined blockade of NEP and angiotensin-converting enzyme (ACE)—to prevent the accumulation of pro-hypertensive angiotensin II—leading to the so-called vasopeptidase inhibitors.^103^ However, in heart failure, the vasopeptidase inhibitor, omapatrilat, did not meet its primary endpoint of all-cause death or hospitalization for HF vs. enalapril but was associated with an increased incidence of angioedema (likely because both NEP and ACE are involved in the degradation of bradykinin).^104^ In hypertensive patients, the effect of omapatrilat on systolic blood pressure change and use of adjunctive antihypertensive therapy exceeded that of an ACE inhibitor alone, but again at the expense of a higher incidence of angioedema.^105^ Accordingly, omapatrilat did not make it to its clinical use.

Co-crystallizing the NEPi sacubitril with the angiotensin II type 1 receptor blocker, valsartan, in a one-to-one molar ratio as LCZ696, jointly termed an angiotensin receptor-neprilysin inhibitor (ARNI), was more successful than valsartan in reducing diastolic blood pressure in hypertensive patients, with no reports of angioedema.^106^ The rationale was to avoid the double hit on bradykinin breakdown and angioedema by blocking angiotensin II type 1 receptors rather than inhibiting ACE. In HFrEF patients, sacubitril-valsartan reduced the risk of cardiovascular death and HF hospitalization more effectively than the ACE inhibitor, enalapril (PARADIGM-HF).^107^ PARAGON-HF compared sacubitril-valsartan vs. valsartan alone in HFpEF, but the primary outcome of total hospitalizations and death from cardiovascular causes did not differ.^108^ A high heterogeneity within the HFpEF population and the definition of HFpEF itself might be the underlying explanations of the failure of PARAGON-HF.^108,109^ Indeed, sacubitril-valsartan was beneficial in a subgroup with lower ejection fraction, a patient population more likely to represent early HFrEF rather than HFpEF.^108^ The protective effect in women remains unclear and warrants further investigation.^108^ The abovementioned studies have led LCZ696 to get FDA approval for HFrEF^110^ and also very recently for HFpEF patients with stronger evidence for those with below-normal LVEF.^111^ LCZ696 is recommended to replace ACE inhibitor as first-line treatment for HFrEF ambulatory symptomatic patients despite optimal therapy with ACE inhibitor, beta-blocker and a mineralocorticoid receptor antagonist according to ESC/AHA guidelines.^10,112^ In addition, a meta-analysis showed a potent antihypertensive effect of sacubitril-valsartan vs. valsartan alone or olmesartan in elderly hypertensives.^113^

## 4. Drugs preventing cGMP breakdown

In addition to enhancing cGMP production, PDE inhibitors can exert, in principle, similar effects by inhibiting cGMP degradation. However, therapeutic exploitation of PDE inhibition has not been as great as one might have anticipated. A total of 11 superfamilies of PDE isoforms are present at different subcellular localizations, thereby targeting different cGMP (or cAMP) enzymatic sources and pools. With respect to cGMP, especially PDE1, 2, 3, 5, and 9 have been implicated in cardiovascular disorders.^114^

### 4.1 PDE5

Sildenafil and tadalafil are used in erectile dysfunction, as first-line treatments in Europe and USA,^13,115^ and in PH; among the initial treatments that can be chosen since there are not available head-to-head comparisons between compounds according to ESC/CHEST guidelines.^11,63^ Sildenafil also improved peak oxygen uptake in PH due to HFrEF^116^ and pulmonary pressure and right ventricular function in PH due to HFpEF.^117^ It showed beneficial effects on glycometabolic control and P-selectin in T2D.^118^ In HFrEF, sildenafil improves left ventricular (LV) diastolic function and cardiac geometry, while in diabetic cardiomyopathy benefits LV contraction.^119,120^ In another use-extension trial in HFpEF, sildenafil showed no improvement in exercise capacity or clinical status.^121^ However, in HFpEF, cGMP concentrations are down-regulated due to low NO bioavailability,^27^ while sildenafil minimally increases plasma cGMP;^121^ thus, PDE5 inhibition would not be expected to represent an effective mechanism-based approach whilst the cGMP dysfunction most likely comes from a source different from the targeted one.

### 4.2 PDE3

The PDE3 inhibitor milrinone is licenced in Europe and USA for acute HF in its intravenous form as second-line treatment,^10,54^ while oral milrinone was associated with increased all-cause and cardiovascular mortality in severe chronic heart failure.^122^ Another PDE3 inhibitor, cilostazol, has antithrombotic properties and, as such, has been under investigation for its antiplatelet effects in T2D (NCT02983214, NCT03248401, NCT02933788). In T2D patients with symptomatic lower extremity artery disease, cilostazol reduced the incidence of acute ischaemic stroke/transient ischaemic attack, acute myocardial infarction, and vascular causes-associated death.^123^ Moreover, in T2D with carotid atherosclerotic plaques, it diminished the carotid plaque progression.^124^ This benefit can be explained mechanistically by a crosstalk between cGMP and cAMP, where cAMP-specific PDE3 is inhibited by cGMP through direct competition at the catalytic site. Thus, some effects of cGMP, e.g. in platelets, are likely to be mediated at least in part via the cAMP-PKA axis.^125^ More recently, PDE3 inhibition was explored in HFpEF, focusing on a new extended-release version of milrinone.^126^ This small pilot study showed a safe profile and improved quality of life in HFpEF patients.^126^

The inotrope-dilator molecule levosimendan is used in 60 countries outside the USA as second-line treatment for acute HF^10,127^ and, in addition to its calcium-sensitizing properties, also inhibits PDE3.^128^ A recent study found that in HFpEF with PH, levosimendan improved the 6-min walk test, although the exercise pulmonary capillary wedge pressure was not significantly reduced.^129^ Important questions remain regarding the population of patients for which this would be beneficial and how mechanism-based patient stratification can be performed.

### 4.3 PDE1

The PDE1 inhibitor, vinpocetine, improved clinical outcomes and reduced lesion size in acute ischaemic stroke through inhibition of NF-κB-dependent inflammation.^130^ This agent, however, is a weak PDE1 inhibitor and also blocks sodium channels and regulates NF-κB signalling.^131^ The novel and potent PDE1 inhibitor, ITI-214, was recently tested in patients with HFrEF (NCT03387215). This double-blind, placebo-controlled multi-dosage trial revealed that ITI-214 induces systemic arterial vasodilation and increases cardiac output and mean LV power.^132^

### 4.4 PDE9A

PDE9A is the most selective cGMP-hydrolysing PDE of the superfamily.^29^ In 2015, a study performed in mice demonstrated a role in a model of cardiac pressure-overload, with both global genetic deletion and treatment with a selective PDE9 inhibitor reducing hypertrophy and fibrosis while improving cardiac function.^29^ The study established a close linkage of PDE9A with the regulation of cGMP generated by NP (rather than nitric oxide) signalling.^29^ In a recent study in mice, the PDE9 inhibitor CRD-733 improved HF characteristics; human trials in HF using CRD-733 are now underway.^133^

### 4.5 PDE10A

PDE10A is a dual cAMP/cGMP PDE. In a recent study in mice, PDE10A inhibition with TP-10 improved pathological cardiac remodelling.^134^ PDE10A inhibition has been clinically tested in schizophrenia and Huntington’s disease, proving that it is a safe target for drug treatment and a potential therapeutic option for diseases related to cardiac remodelling.^134^

## 5. Network pharmacology

As indicated above, cGMPopathies can emerge from different dysfunctions within cGMP formation, breakdown or signalling. Network medicine analysis, however, shows that the specific cGMP source matters. PPI networks of validated seed genes suggest that cGMP signalling is segregated into modules. These modules are likely to define the therapeutic (and diagnostic, see below) targets. Thus, NP analogues may not necessarily compensate for a loss of GC-1/2 function, while *vice versa*, sGC stimulators may not compensate for a loss of GC-A or GC-B-mediated cGMP production; a phenomenon exemplified in experimental heart failure.^135^

**Figure 3:**
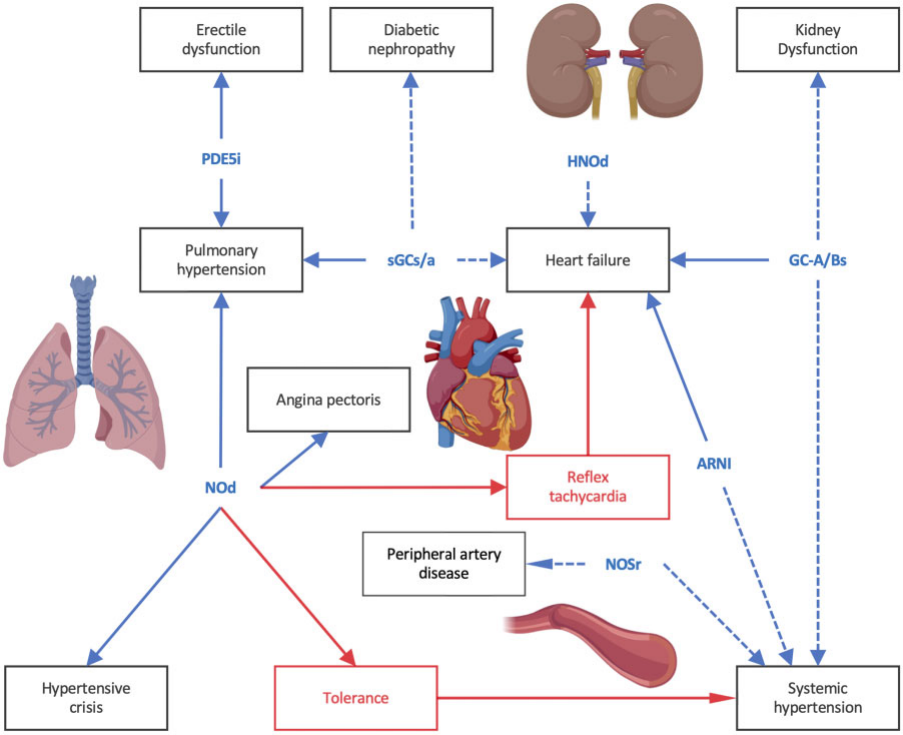
cGMPopathies and evidence-based therapies. Potential combinatorial cGMP therapies in cardiovascular applications. Blue dashed lines show drugs in clinical trials, whereas solid blue lines approved drugs. Red lines show limitations of usage in these indications. ARNI, angiotensin receptor-neprilysin inhibitor; GC-A/Bs, GC-A/B stimulators; NOd, NO donors; HNOd, nitroxyl donors; NOSr, NOS recoupling drugs; PDEi, PDE inhibitors; sGCs/a, sGC stimulators/activators. Created with BioRender.com.

A complex disease mechanism is comprised of a protein network rather than being definable by a single target protein. Within these network modules, the specific source of cGMP matters, as they are not interchangeable. Moreover, another important therapeutic option emerges from that i.e. network pharmacology. A dysfunctional multi-protein network is more likely to be remedied to a more physiological state by several drugs targeting different proteins of the same module. This should occur in a synergistic manner allowing reductions in the dose of each individual drug whilst retaining overall efficacy but likely reducing side effects.^136^ This mechanism-based network pharmacology approach is different and not to be confused with classical combination therapy, where drugs are combined that have different mechanism of action, none of which is causal for the disease, and effects are maximally additive. A clinical application of this approach is a triple-drug combination in patients with cystic fibrosis; these drugs target a causal mechanistic dysfunction increasing the eligibility for up to 90% of cystic fibrosis patients.^137^ Since cystic fibrosis has cardiovascular symptoms or phenotypes,^138^ this is also a good example for both network pharmacology and an organ-agnostic approach to disease. With respect to cGMPopathies, one approach may be to enhance cGMP production and at the same time inhibit cGMP degradation,^139^ but such combinations have to be chosen in an evidence-based manner, strictly within one disease module (*Figure 3*). Preclinical models indicate that PDE5 is more involved in regulating GC-1/2 signalling (e.g. in erectile dysfunction), whereas PDE9 and PDE2 are biased towards NP signalling (e.g. in heart failure).^29,140^ It remains to be seen whether some of the negative clinical studies (e.g. cinaciguat and sildenafil in heart failure) can be explained by the use of a suboptimal cGMP-modulating therapy.^77,121^ However, such combinations may also be contraindicated in one setting and indicated in another. A possible example of this is sildenafil and NO donors, which are contraindicated as they lead to severe hypotension.^141^ A similar excessive decrease in systemic blood pressure was observed with riociguat and sildenafil in patients with PH (PATENT PLUS trial).^142^ Conversely, a small (six patients), pilot clinical trial investigating a combination of the tried-and-tested organic nitrate, ISMN, and the PDE5 inhibitor, sildenafil, appeared to achieve better regulation of the blood pressure in patients afflicted with ‘resistant’ hypertension.^143^ Another proof-of-concept example came from the phase IIa trial of neprilysin inhibition in PAH patients already stable on PDE5i; this mechanistic combination seems to have an additional benefit in PAH.^144^

## 6. How to diagnose cGMPopathies and stratify patients?

**Figure 4:**
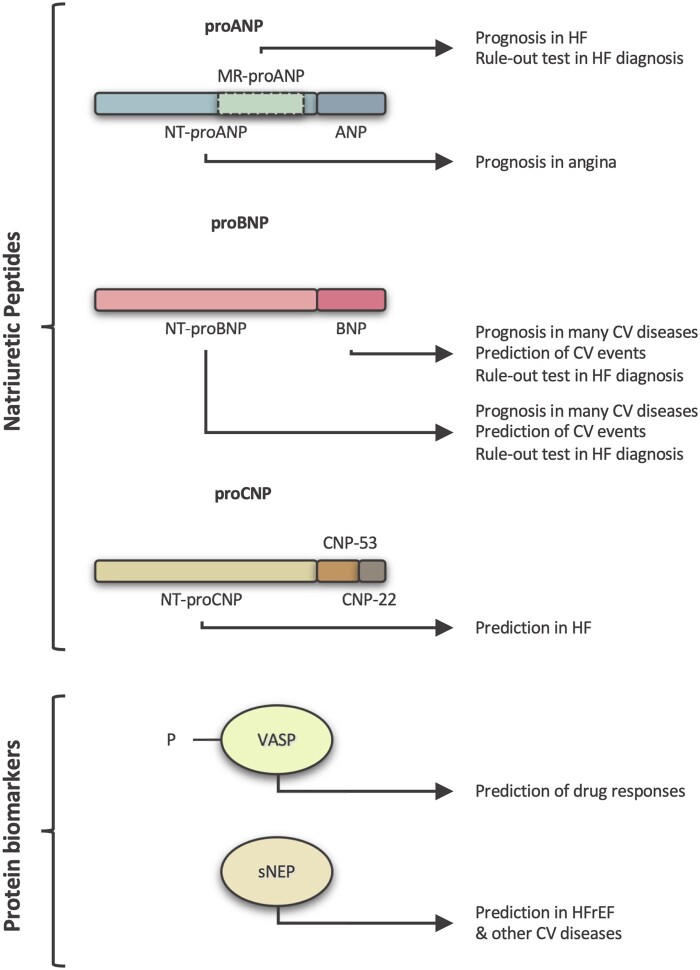
cGMP diagnostic toolbox. cGMP-related biomarkers currently used in the clinic for cardiovascular diseases. NPs and their precursors, sNEP and P-VASP have several diagnostic, prognostic or predicting applications in cardiovascular disorders. CV, cardiovascular.

The missing link between cardiovascular phenotypes and cGMP-modulating treatments are mechanism-based biomarkers. Such tools would identify the patients that have a cGMPopathy and also the specific part of the pathway that should be targeted. Therefore, who would benefit from a cGMP therapy and which cGMP-targeting drug or drugs combination to choose remain unknown. Here, we review the current cGMP-related biomarkers and their applications. In principle, three approaches exist to assess endogenous cGMP signalling in patients: (i) cGMP itself, (ii) cGMP-PKG-dependent protein phosphorylation (e.g. of the vasodilator-stimulated phosphoprotein, VASP); or (iii) levels of endogenous GC stimulators (NO- or NP-related). Of clinical relevance, so far, are only circulating NPs and phospho-VASP (*Figure 4*).

### 6.1 cGMP

cGMP has been used to monitor drug-induced increase as proof of target engagement e.g. with ARNis, designer NPs, and PDE-5 inhibitors.^145–147^ However, variation in cGMP concentrations between individuals has hindered its use as a biomarker for primary diagnosis.^148^

### 6.2 P-VASP

P-VASP was introduced two decades ago as a new biomarker able to monitor the vascular NO/cGMP/PKG signalling.^149^ In principle, cell-based assays could be suited to detect defective endogenous cGMP signalling, e.g. via lower than normal phosphorylation of VASP or other PKG target proteins. However, both the phosphorylation and dephosphorylation kinetics^150^ would require extremely reproducible procedures with respect to blood collection, work-up and analysis. So far, this has prevented the establishment of basal P-VASP levels as a biomarker. In contrast, P-VASP assays are clinically established to assess drug responses, e.g. to predict responders and non-responders to antiplatelet drugs to reduce major cardiovascular and cerebral events.^151,152^ P-VASP responses to sGC activators have been used to detect a higher apo-GC-1/2/Fe(II)GC-1/2 ratio in CAD patients,^37^ which could be used for mechanism-based therapy in patients with elevated apo-GC-1/2 levels.

### 6.3 NPs and soluble neprilysin

Each of the NPs has been proposed as a predictive biomarker for cardiovascular diseases or to guide cardiovascular therapy. The best characterized is BNP and its N-terminal fragment post-processing NT-proBNP. The lack of NT-proBNP degradation by NEP and analogous enzymes makes it superior to BNP for monitoring patients. Plasma levels of BNP, NT-proBNP, and mid-regional pro-atrial natriuretic peptide (MR-proANP) have been used to aid the heart failure diagnosis as ‘rule-out’ tests, excluding significant cardiac dysfunction.^10^ Of note, the 2016 *ESC Guidelines on the Management of Acute and Chronic Heart Failure* recommend the use of BNP and NT-proBNP for the diagnosis of HF.^10^ Furthermore, BNP levels are associated with poor prognosis in stroke,^153^ NT-proBNP levels in hypertrophic cardiomyopathy,^154^ while both peptides predict cardiovascular events in the general population,^155^ and poor outcome in heart failure.^156,157^ However, NPs do have some limitations as diagnostic markers since there are many confounding factors.^10^ Indeed, age, sex, renal function but also cardiovascular diseases, including volume expansion and possibly increased wall stress, ischaemia, and hypertension, all affect circulating NP concentrations.^10,158^

N-Terminal pro C-Type Natriuretic Peptide predicts HFpEF outcome^159^ and is negatively associated with myocardial ageing;^160^ N-terminal pro ANP has been proposed as a prognostic biomarker in stable angina;^161^ MR-proANP, in heart failure.^162^ Finally, high levels of circulating soluble NEP (sNEP) predict outcome in HFrEF,^163^ but not HFpEF,^164^ diabetes and other cardiovascular diseases.^165^ Application of the above correlations for NP-guided therapy is less developed and its value uncertain: HF-related hospitalization may be reduced^166^ and patients with cardiovascular risk factors but not heart failure may benefit.^167^

### 6.4 Nitrogen oxides (NOx)

Many pathological conditions have been associated with altered levels of nitric oxide through measurement of more stable metabolites, nitrite and nitrate (collectively abbreviated NOx) or nitrotyrosine. Rapidly measured plasma nitrite rather than nitrate reflects endothelial nitric oxide synthase activity.^168^ Nitrotyrosine, either scavenging of nitric oxide through ROS or myeloperoxidase activity, is associated with increased inflammation.^169^ Lower levels of NOx are associated with a more severe outcome in stroke and with increased mortality in idiopathic PAH,^170,171^ while higher levels of NOx and nitrotyrosine correlate with increasing severity of chronic HF.^169^ Finally, higher levels of NOx correlate with cardiovascular mortality.^172^

### 6.5 Asymmetric dimethylarginine (ADMA)

Endogenous ADMA and N^G^-monomethyl-L-arginine (L-NMMA) attenuate L-arginine-dependent NO production by inhibiting and uncoupling NOS.^173^ Elevated levels of ADMA impair endothelial function and thus promote atherosclerosis.^174^ ADMA and L-NMMA are possibly strong and independent risk factors for cardiovascular disorders, such as hypertension, CAD, atherosclerosis, PH, atrial fibrillation, stroke, and peripheral artery disease.^173,175^ However, ADMA-guided interventional studies are missing.

## 7. Summary and outlook

Several cGMP-modulating drugs have entered the clinical arena with indications across a wide spectrum of cardiovascular disease states. Based on genetic evidence,^18,19^ correcting dysfunctional cGMP signalling, i.e. cGMPopathies, has the potential to become one of the few mechanism-based, causal interventions in cardiovascular medicine. Whilst all necessary drugs seem to be available, the key challenge will be to identify those patients with the right indications that present not only a suitable phenotype but, importantly, also exhibit cGMP dysfunction, i.e. the mechanotype. Some of the recent failures in HFpEF drug development may have been preventable by mechanism-based patient stratification. PKG phosphoprotein panels in combination with markers, such as ADMA and nitrotyrosine, may be components of such a cGMPopathy diagnostic algorithm. Once this milestone is achieved, diagnostic-enabled cGMP precision therapy will be possible, most likely by network pharmacology i.e. using multiple cGMP-modulating drugs with different targets in a synergistic manner and in doses that are lower than single drug approaches and, consequently, lower side effects. Based on the compartmentalization of cGMP and unique functions, there is a rationale for further drug discovery on both sGC and pGC. Moving from reductionistic approaches of disease development to molecular network modules is vital to understand the underlying mechanism of a disease state and the connection with its comorbidities, which is one of the reasons preclinical research fails to be translated in the clinic.^176,177^ Clearly, we are in an era of increasing clinical relevance and high precision, mechanism-based and curative applications of cGMP-modulating drugs.
